# Role of CD40(L)-TRAF signaling in inflammation and resolution—a double-edged sword

**DOI:** 10.3389/fphar.2022.995061

**Published:** 2022-10-04

**Authors:** Lea Strohm, Henning Ubbens, Thomas Münzel, Andreas Daiber, Steffen Daub

**Affiliations:** ^1^ Department of Cardiology, Cardiology I—Laboratory of Molecular Cardiology, University Medical Center of the Johannes Gutenberg-University Mainz, Mainz, Germany; ^2^ German Center for Cardiovascular Research (DZHK), Partner Site Rhine-Main, Mainz, Germany

**Keywords:** CD40(L)-TRAF signaling, inflammation, resolution, cardiovascular disease, ischemia, reperfusion

## Abstract

Cardiovascular diseases (CVD) and cardiovascular risk factors are the leading cause of death in the world today. According to the Global Burden of Disease Study, hypertension together with ischemic heart and cerebrovascular diseases is responsible for approximately 40% of all deaths worldwide. The major pathomechanism underlying almost all CVD is atherosclerosis, an inflammatory disorder of the vascular system. Recent large-scale clinical trials demonstrated that inflammation itself is an independent cardiovascular risk factor. Specific anti-inflammatory therapy could decrease cardiovascular mortality in patients with atherosclerosis (increased markers of inflammation). Inflammation, however, can also be beneficial by conferring so-called resolution, a process that contributes to clearing damaged tissue from cell debris upon cell death and thereby represents an essential step for recovery from, e.g., ischemia/reperfusion damage. Based on these considerations, the present review highlights features of the detrimental inflammatory reactions as well as of the beneficial process of immune cell-triggered resolution. In this context, we discuss the polarization of macrophages to either M1 or M2 phenotype and critically assess the role of the CD40L-CD40-TRAF signaling cascade in atherosclerosis and its potential link to resolution. As CD40L can bind to different cellular receptors, it can initiate a broad range of inflammatory processes that may be detrimental or beneficial. Likewise, the signaling of CD40L downstream of CD40 is mainly determined by activation of TRAF1-6 pathways that again can be detrimental or beneficial. Accordingly, CD40(L)-based therapies may be Janus-faced and require sophisticated fine-tuning in order to promote cardioprotection.

## 1 Introduction

Cardiovascular diseases (CVD) are classified as inflammation-related entities (Bartekova et al., 2018). Thus, it is not surprising that diseases associated with systemic inflammation, such as rheumatoid arthritis (Bergholm et al., 2002; Hänsel et al., 2003), psoriasis (Mehta et al., 2010), or type-2 diabetes (Henry et al., 2004), contribute to increased cardiovascular risk. Endothelial dysfunction occurs, for example, in severe inflammation triggered by lipopolysaccharide (LPS) (Becker et al., 2012). In 2005, the research group around Ridker could show that death caused by myocardial infarction (MI) in patients with the acute coronary syndrome can be predicted by a high CRP (C-reactive protein) level (Ridker et al., 2005). A correlation between inflammation and an increased risk of CVDs, but also between inflammation, redox reactions, and oxidative stress is evident (Karbach et al., 2014; Steven et al., 2015; Daiber et al., 2017b). However, inflammation plays a major role not only in its interaction with CVDs, as its influence can also be shown in neurodegenerative diseases such as Alzheimer´s (Prasad, 2016; Wes et al., 2016; Katsumoto et al., 2018) or Parkinson’s disease (Gorelenkova Miller et al., 2016; Troncoso-Escudero et al., 2018). Therefore, diseases associated with inflammation represent an important area of research, as they are major challenges, both for our society and our health system.

### 1.1 Inflammation

Inflammation plays a vital role in CVD and chronic diseases such as fatty liver disease, kidney disease, and neurodegenerative diseases are triggered by or via the interaction with inflammation (Steven et al., 2015; Daiber et al., 2017a; Daiber et al., 2017b). Oxidative stress is characteristic of CVD and is mainly associated with inflammation (Harrison et al., 2011; Karbach et al., 2014; Wenzel et al., 2017). This represents a risk factor in CVD (Kaptoge et al., 2014) that can be counteracted with pharmacological agents (Ridker et al., 2017). All the evidence suggests that a correlation exists between chronic inflammatory (e.g., lupus erythematosus, psoriasis, rheumatoid arthritis, autoimmune diseases) and cardiovascular diseases, as they show an increased cardiovascular risk (Hak et al., 2009; Vena et al., 2010; Soltész et al., 2011; Murdaca et al., 2012). Of particular note, psoriasis has been highlighted as an independent factor in CVDs (Mehta et al., 2010). The European Alliance of Associations for Rheumatology (EULAR) recommends rigorous screening for cardiovascular disease and an early, intensive therapeutic approach in patients who have psoriasis or rheumatoid arthritis (Peters et al., 2010). This recommendation results from the fact that endothelial dysfunction is observed in both patient groups (Balci et al., 2009; Södergren et al., 2010). In line with the immunosuppressive therapy approach, further studies have shown that targeted therapy reduced cardiovascular mortality in rheumatoid arthritis (IL-6, TNF-α, and IL-17A cascades) (Pasceri and Yeh, 1999; Choy, 2012), lupus erythematosus (IL-17A signaling) (Crispín and Tsokos, 2010), and psoriasis (IL-17/IL-23) (Di Cesare et al., 2009; Papp et al., 2012). The cytokines mentioned above (interleukins IL-6, IL-1β, and IL-17A, as well as tumor necrosis factor-α [TNF-α]) should be considered in a positive relationship with CVD (Karbach et al., 2014).

In this regard, there are clinical trials with receptor blockers or antagonists being conducted or completed in patients with CVD (van Tassell et al., 2013). Likewise, a study from the Genome-Wide Association Study (GWAS) showed that inflammatory processes play a significant role in both the development and progression of CVDs. For example, point mutations were found in or near genes involved in cell adhesion, leukocyte migration and atherosclerosis (PECAM1, rs1867624), inflammation, and coagulation (PROCR, rs867186 p. Ser219Gly) (Howson et al., 2017). In addition, further GWAS studies identified the association of several risk loci with cardiovascular inflammation (Klarin et al., 2017). Upon stress-dependent activation (e.g., inflammatory stimuli) or apoptosis of endothelial cells, they release endothelial microparticles (EMPs). These vesicles trigger oxidative stress and can lead to inflammation and eventually promote the development of atherosclerosis (Paudel et al., 2016). Immunosuppressive and anti-inflammatory properties of cardiovascular drugs such as AT1R blockers or ACE inhibitors and antidiabetic drugs such as GLP-1 receptor agonists or DPP-4 inhibitors extend beyond the drugs’ traditional therapeutic approach (Shah et al., 2011; Matsubara et al., 2012; Steven et al., 2015). Another example for the efficacy of specific anti-inflammatory therapies in CVD was shown in the CANTOS trial, in which the IL-1β antagonist canakinumab was used and improved cardiovascular outcome in a dosage-dependent manner (Iseme et al., 2017) through inhibition of the IL-1β pathway and consequent reduction in CRP levels. The use of canakinumab in patients with diabetes mellitus and additional cardiovascular disease indicate a positive outcome (Karbach et al., 2019). In summary, these findings suggest causality between CVD, and autoimmune disease *via* inflammation and demonstrate the possibility of an anti-inflammatory therapeutic approach to cardiovascular disease (Vena et al., 2010; Soltész et al., 2011).

### 1.2 Oxidative stress

The fact that oxidative stress plays a pivotal role in the development of CVD was shown by Harrison and Ohara as early as the 1990s using the experimental model of hypercholesterolemia (Ohara et al., 1993; Harrison and Ohara, 1995). Since then, it has been understood that most CVDs exhibit a mismatch between ROS (reactive oxygen species) formation and their degradation by antioxidant mechanisms (Heitzer et al., 2001; Griendling and FitzGerald, 2003). This mismatch leads to the accumulation of superoxide, peroxynitrite, hydrogen peroxide, and hypochlorous acid and puts the system into disequilibrium (Sies, 2015). Our research group has detailed the underlying molecular mechanisms of vascular dysfunction—the ROS sources involved, redox mechanism in the -NO/cGMP pathway (Daiber et al., 2014; Schulz et al., 2014; Wenzel et al., 2017). On the other hand it is also possible to initiate ROS production *via* crosstalk of involved activation pathways (Daiber, 2010; Dikalov, 2011; Daiber et al., 2017a). For example, this mechanism has been described for the NADPH oxidase isoform 2 (NOX-2)/mitochondrial axis (Doughan et al., 2008; Dikalova et al., 2010), xanthine dehydrogenase/oxidase conversion, and eNOS (endothelial nitric oxide synthase) uncoupling (Kröller-Schön et al., 2014; Schulz et al., 2014). The principle of redox crosstalk has also been observed in angiotensin II (AT-II)-induced hypertension: Here, activation of NOX-2 leads to Sirt3 S-glutathionylation, which contributes to acetylation of SOD2 (superoxide dismutase type 2, mitochondrial isoform) and thus decreases the enzyme’s antioxidant activity. This circumstance, in turn, increases mitochondrial superoxide and reduces nitric oxide bioavailability in the endothelium, thus aggravating hypertension (Dikalova et al., 2017; Dikalov and Dikalova, 2019). Likewise, this redox crosstalk has been associated with tobacco use and its vascular effects (Dikalov et al., 2019).

Furthermore, the uncoupling of eNOS was shown to represent a key step in the formation and progression of CVDs (Schulz et al., 2014; Münzel et al., 2005; Förstermann and Münzel, 2006). Numerous studies have shown that the interplay between oxidative stress and cardiovascular disease can be used in order to ascertain a patient’s prognosis. As an example, a positive correlation was shown between glutathione peroxidase-1 and outcomes in patients with cardiovascular disease (Blankenberg et al., 2003). Various clinical studies have shown that CVD patients have an increased blood-level of the oxidative DNA damage marker 8-hydroxy-2-deoxyguanine, which can be used as a surrogate parameter for oxidative stress (Di Minno et al., 2016). Despite these lines of evidence for an association of oxidative stress markers with CVD, large-scale clinical trials that support a beneficial impact of antioxidant therapy on the prognosis of CVD patients are lacking (Münzel et al., 2010; Schmidt et al., 2015). Finally, one can postulate that there is much evidence for the negative impact of oxidative stress on physiological and inflammatory processes (Wenzel et al., 2008; Oelze et al., 2014).

## 2 Resolution in myocardial infarction

**FIGURE 1:**
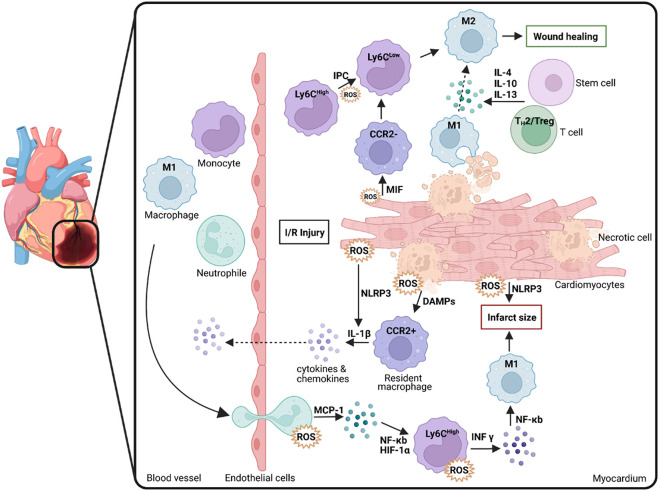
Macrophage polarization, infiltration and ROS generation following myocardial damage (e.g., through I/R injury). Macrophages are crucial in the process underlying tissue inflammation as well as wound healing and inflammatory resolution thereby determining the degree of infarct size. M1: macrophages with M1 polarization; Ly6C: Lymphocyte antigen 6 complex locus C; M2: macrophages with M2 polarization; IL: Interleukin; CCR2: C-C chemokine receptor type 2; ROS: Reactive oxygen species; IR: Ischemia/reperfusion; MIF: Macrophage migration inhibitory factor; NLRP3: Family pyrin domain containing 3; DAMP: Damage-associated molecular pattern; MCP: Monocyte Chemoattractant Protein-1 (CCL-2); NF-κB: Nuclear Factor Kappa B; HIF-1α: Hypoxia inducible factor 1 alpha; INF-γ: Interferon gamma; IPC: Ischemic preconditioning, Treg: Regulatory T cells; T_H_1/2: T Helper cells 1/2. Scheme was summarized from data in (Daiber et al., 2021). Created with BioRender.com.

In addition to the factors influencing inflammation and its resulting diseases discussed so far, seemingly mundane factors such as unbalanced sleep (Arnett et al., 2019), poor diet, or lack of exercise are also important. They can influence molecular processes in the body and negatively affect the immune system (Frodermann et al., 2019; McAlpine et al., 2019; Tourki and Halade, 2021). Since the development of inflammation plays a vital role in CVDs, it is crucial to focus on its processes to develop strategies to treat CVDs (Murphy et al., 2020). In the disease course of CVDs, a distinction is made between mild, acute, and chronic inflammation. In patients suffering from diabetes or metabolic disorders, mild inflammation that occurs within the course of the underlying disease can become chronic if inflammatory resolution is not achieved (Fuster et al., 2016). For example, in MI, inflammation is crucially involved in plaque formation as well as plaque rupture and subsequent tissue hypoxia. The severity of the resulting ischemic heart failure (HF) depends strongly on the resolution of the inflammation, which is part of the healing process (Figure 1). This active process runs in parallel with inflammation and ensures the degradation of the resulting inflammatory products (Serhan, 2011; Serhan, 2014). Resolution-promoting lipid mediators (SPMs) are released in this process, and pro-inflammatory cells are differentiated into an immunoreactive phenotype (Halade et al., 2017; Kain et al., 2017; Sharma et al., 2020).

Immediately after the onset of MI, leukocytes are dispatched to the heart to ablate damaged tissue there, ultimately effecting the inflammatory process (Halade et al., 2018). In addition, specific cytokines/chemokines, inflammatory and endothelial cells, and ROS are produced or activated (Tracy, 2006). However, the subsequent resolution or its successful progression is age-dependent, as there is increased dysregulation between pro- and anti-inflammatory molecules and subsequent pathways in older patients (Lopez et al., 2015; Halade et al., 2016). The first immune cells to respond to inflammation are neutrophils, which counteract stress-induced cell dysfunction (Kain and Halade, 2020). In inflammation and resolution, neutrophils appear according to a chronological polarization pattern in differentiated phenotypes with correspondingly divergent functions, which are subdivided as pro-inflammatory N1 and anti-inflammatory N2 neutrophils and whose role includes both tissue repair but also precisely damage to unaffected surrounding tissue (Vafadarnejad et al., 2020). At the onset of MI, N1 neutrophils are active and are subsequently replaced by N2 neutrophils, which are anti-inflammatory and enact tissue repair. In addition, monocytes belonging to the domain of leukocytes and are derived from the bone marrow circulate in the organism for up to 3 days (Duncan et al., 2020). These monocytes can differentiate into macrophages, which play an essential role in inflammation and repairing damaged tissue. Depending on phenotypic plasticity and polarization, macrophages are also divided into pro-inflammatory M1 and anti-inflammatory M2 macrophages and influence the healing process, differentiation of myofibroblasts from fibroblasts, degradation of cellular debris, tissue repair through recruitment of inflammatory cytokines/chemokines, and activation of endothelial cells (Duncan et al., 2020) (Figure 1). According to a recent study by Chiurchiù and colleagues, macrophages are a significant factor in the progression of resolution, such as regulatory T lymphocytes (Tregs) which limit pro-inflammatory responses of macrophages and T lymphocytes in their function which in turn places macrophages in an anti-inflammatory state that supports tissue repair. In addition, lymphocytes from patients with chronic heart failure have been shown to exhibit impaired immune system interference-mediated via CD8^+^ and CD4^+^ cells, indicative of impaired resolution (Chiurchiù et al., 2019).

Previously, the resolution of inflammation was thought to be a largely passive process in which inflammatory factors, enzymes involved, and signaling pathways were inhibited or blocked (Serhan, 2011). However, recent studies show a specific resolution pathway mediated by bioactive mediators (resolution-promoting mediators) (Gerlach et al., 2020). This resolution pathway is determined by several regulatory factors, such as resolution-promoting lipids (e.g., protectin, lipoxin, resolvin) and neutrophils, T cells, and macrophages, each of which is converted to its anti-inflammatory phenotype (Halade et al., 2017; Kain et al., 2017; Sharma et al., 2020). In this resolution process, the secretion of pro-inflammatory cytokines (IL-6, TNF-α) is throttled, the production of anti-inflammatory cytokines (IL-10) is stimulated, neutrophils infiltration into the tissue reduced, and phagocytic macrophages are stimulated. This resolution pathway may represent a starting point that positively influences the prognosis of CVDs. The prerequisite for a favorable prognosis after an inflammatory MI is the restoration of systemic homeostasis through the repair of the damaged tissue. In this process, macrophages play essential roles by secreting anti-inflammatory cytokines such as transforming growth factor-beta (TGF-β) or vascular endothelial growth factor (VEGF) (Ortega-Gómez et al., 2013). These cytokines are instrumental in regenerating damaged tissue, ultimately promoting homeostasis (Ortega-Gómez et al., 2013).

### 2.1 Macrophages—M1 vs. M2

MI leads to ischemia of the myocardium in the medium to long term, resulting in the death of the muscle tissue. MI represents one of the most common causes of disease-related death (Sadek and Olson, 2020). As a result of the undersupply of oxygen to the tissue, after MI, necrosis occurs, triggering both systemic and local inflammation. Neutrophils and monocytes migrate into the damaged tissue and start the repair mechanism, divided into 3 phases described in detail in the preceding sections, inflammation followed by repair of the tissue, and finally, healing and resolution (Forte et al., 2018). While a plethora of macrophage phenotypes that are involved in the formation and progression of atherosclerosis have been recognized, including Mhem, Mox, and M4, the following section will focus on two extreme ends of the spectrum, namely M1 and M2 macrophages.

To limit tissue necrosis due to oxygen insufficiency and thus improve prognosis in MI, it is time-critical to reverse ischemia by reperfusion. This ischemia/reperfusion (I/R) can be achieved, for example, by pharmaceutical thrombolysis or mainly mechanical intervention, such as percutaneous coronary intervention (PCI), in which the occluded vessel can resume blood supply to the tissue using angioplasty followed by stent implantation (Vincent et al., 2017). In addition, macrophages are known to play a crucial role in the course of ischemic MI with subsequent reperfusion (Vagnozzi et al., 2020), which is related to their pro- and anti-inflammatory phenotype.

There is a broad consensus that the M1 phenotype of macrophages, especially at the onset of I/R, further increases the damage by secreting inflammatory factors and proteases and producing ROS (Formigli et al., 2001; Steffens et al., 2009). However, recent studies demonstrate that phagocytosis performed by M1 macrophages plays a vital role in subsequent tissue repair (Figure 1). M1 macrophages predominate in the myocardium and are mainly polarized by the transmembrane receptor dectin-1, leading to ROS formation but also to NF-κB activation and subsequent secretion of pro-inflammatory cytokines and chemokines (CCL5, IL-1β, IL-6, IL-12, MCP-1, TNF-α), which worsens the condition after I/R (Fan et al., 2019; Leuti et al., 2021). Diametrically, a previous study has shown that the soluble receptor for glycation end products positively affects cardiac function in the mouse in a model for MI and subsequent I/R by promoting infiltration of M1 macrophages into the damaged tissue and IFN-γ secretion (Zhang et al., 2019). This fact suggests a certain ambivalence to M1 macrophages: While they participate in tissue protection in acute ischemia, they also have the capacity to significantly damage the tissue by secreting pro-inflammatory cytokines and recruiting immune cells.

**FIGURE 2:**
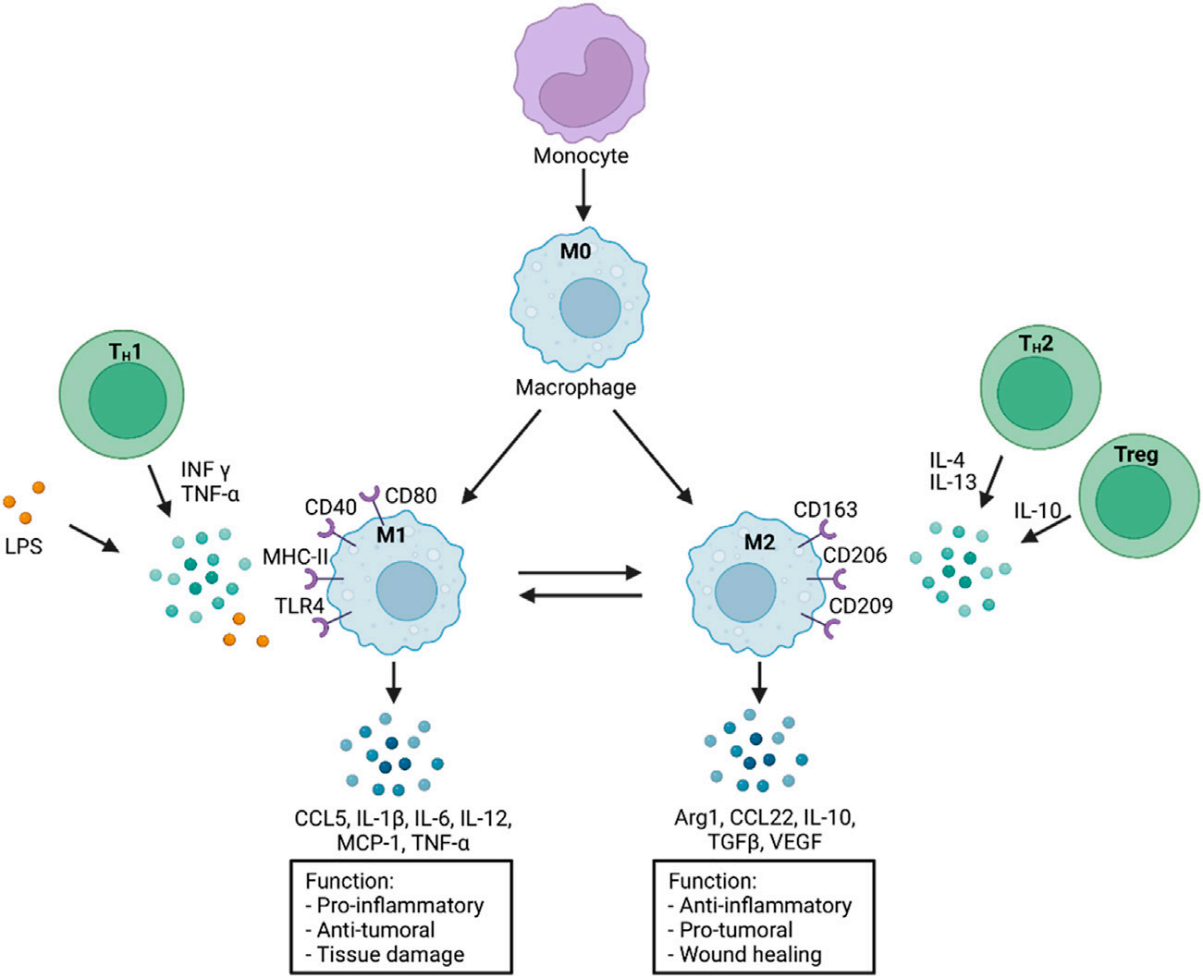
Monocyte differentiation and macrophage polarization in response to various stimuli. A selection of distinct functions is presented on the bottom. M0: macrophages without polarization; INF-γ: Interferon gamma; TNF-α: Tumor necrosis factor alpha; LPS: Lipopolysaccharide; CD: Cluster of differentiation; MHC-II: Major histocompatibility complex-II; TLR4: Toll like receptor 4; CCL5: CC-Chemokine ligand 5; MCP-1: Monocyte chemoattractant protein-1 (CCL2); IL: Interleukin; TGFβ: Transforming growth factor β; Arg1: Arginase 1; VEGF: Vascular endothelial growth factor; Treg: Regulatory T cells; T_H_1/2: T Helper cells 1/2. Scheme was summarized from data in (Yao et al., 2019; Daiber et al., 2021). Created with BioRender.com.

In contrast to M1 macrophages, M2 macrophages, which are polarized by type 2 T helper (T_H_2) cell cytokines (IL-4, IL-13), can secrete anti-inflammatory cytokines (IL-10, TGFβ) and anti-fibrogenic factors that promote a favorable prognosis through their anti-inflammatory and reparative functions (Figure 2) (Leuti et al., 2021). Another study has shown that M2 macrophages alternatively polarized by retinoic acid receptor responder protein 2 (RARRES2) can protect against damage in I/R. This study was performed in mice, and it was observed that pro-inflammatory cytokines were inhibited while the level of anti-inflammatory IL-10 was increased (Chang et al., 2015). A macrophage subtype has been in focus for some time, the regulatory M2b macrophages. Clinical studies have shown that after I/R due to MI, injecting the M2b subtype into the damaged tissue improved myocardial function and reduced cardiac fibrosis (Yue et al., 2020b). Another observation shows that this subtype is not directly involved in the healing process. However, its phenotypic expression modulates the inflammatory immune response and eventually supports a protective and tissue-building effect after I/R (Yue et al., 2020a).

### 2.2 Macrophages in focus to find novel therapeutic targets for chronic inflammatory diseases

As mentioned before, macrophage recruitment as well as the differentiation of M1 and M2 macrophages is a central anchor point in the development of many chronic inflammatory diseases. For this reason, many scientists focus on macrophages to find a possible new therapeutic target to suppress inflammation. It was shown that human placental mesenchymal stem cells (pMCS) could affect macrophage function as well as the differentiation of human monocytes from M1 macrophages to anti-inflammatory M2 macrophages and are therefore potential targets for cell-based therapy. Accompanying the phenotypic shift by pMCSs, the pattern of protein expression and secretion is modulated, and typical M1 macrophage proteins (CD40), chemokines (MIP-1α), and cytokines (IL-1β and IL-12) are significantly downregulated in addition the phagocytic activity of apoptotic cells is upregulated after the differentiation towards M2 macrophages (Abumaree et al., 2013). In another approach BML-111 an analog of lipoxin4, which is an anti-inflammatory lipid mediator, was used to resolve neuroinflammation after ischemic stroke. The infarct size, as well as M1 CD40^+^ macrophage content, was reduced 1 week after stroke and daily injections of BML-111 in rats (Hawkins et al., 2017). Moreover, 48 h after stroke and BML-111 treatment, reduced levels of pro-inflammatory chemokines (MIP-1α and MCP-1) and cytokines (TNF-α and IFN-γ), as well as increased levels of anti-inflammatory cytokines (IL-4 and IL-10), were observed. In a third study, activated macrophages of rheumatoid arthritis patients were treated with anti-TNF-α agents, which lead to decreased production of pro-inflammatory cytokines (TNF-α, IL-6, IL-12), and decreased expression of inflammation marker (CD40), enhanced phagocytic activity as well as polarization towards M2 phenotype. The main driver of the alternative macrophage polarization after TNF-α treatment was the IL10/STAT3 signaling pathway (Degboé et al., 2019). TNF-α inhibitors are already successfully used in the treatment of Chron’s disease or rheumatoid arthritis (Gerriets et al., 2022). Another study analyzed the influence of resolvin D1 *in vitro* as well as in a liver I/R injury model in mice. It was shown that resolvin D1 has anti-inflammatory and pro-resolving properties and leads to enhanced M2 macrophage polarization, which was accompanied by reduced INF-γ, TNF-α, and IL-6 expression but increased IL-4 and IL-10 expression (Kang and Lee, 2016). Taken together pMCSs, cys-SPMs, BML-111 and resolvin D1 could be potential novel therapeutic targets for chronic inflammatory diseases.

## 3 CD40L signaling cascade in atherosclerosis and its link to resolution

In the last decades the CD40-CD40L signaling cascade became recognized as an important player in inflammatory and autoimmune diseases like atherosclerosis, diabetes type 1, rheumatoid arthritis, lupus erythematosus, and allograft rejection (Karnell et al., 2019). The signaling cascade is crucial for the development of inflammatory processes and the generation as well as stabilization of arterial thrombi (Michel et al., 2017; Daub et al., 2020).

### 3.1 Non-classical CD40L-integrin signaling

**FIGURE 3:**
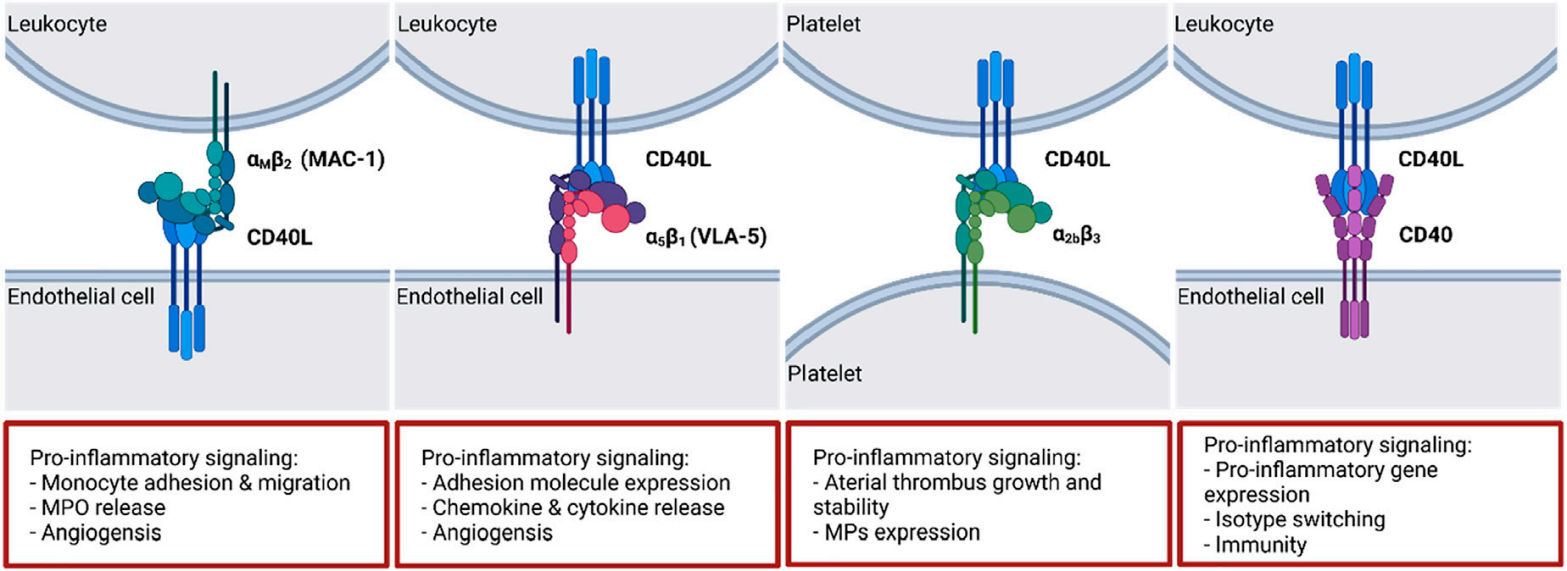
CD40(L) and its ligands and receptors. While CD40L has multiple binding partners, CD40 is largely restricted to ligation by CD40L. The dyad regulates multiple crucial biological processes including isotype switching, thrombus stabilization and cell activation. A selection of typical effects elicited by the interactions is presented on the bottom. Α_M_β_2_: Macrophage antigen 1 (MAC-1); α_5_β_1_: Fibronectin receptor (also: Very late antigen 5 (VLA-5)); α_2b_β_3_: Integrin α_2b_β_3_. Scheme was summarized from data in (Aloui et al., 2014; Michel et al., 2017). Created with BioRender.com.

CD40L (or CD154, 39 kDa protein) belongs to the tumor necrosis factor (TNF) family and is expressed on platelets, activated T lymphocytes, vascular smooth muscle cells, endothelial cells, monocytes, and macrophages. CD40L appears as a surface expressed protein as well as a soluble protein (sCD40L), which is mainly secreted by platelets (Foy et al., 1996; Michel et al., 2017). Four different CD40L receptor proteins are described in the literature (Figure 3): the integrins α_IIb_β_3_, α_5_β_1_ (or VLA-5), and α_M_β_2_ (or Mac-1), as well as CD40 (Bosmans et al., 2021). It was shown that CD40L binding to integrin receptor Mac-1 mediates monocyte adhesion, migration as well as myeloperoxidase (MPO) secretion. Inhibition of Mac-1 *in vivo* leads to reduced macrophage accumulation in atherosclerotic lesions, which slows down the progression of atherosclerosis (Zirlik et al., 2007). Binding of sCD40L to platelet integrin α_IIb_β_3_ mediates stabilization of arterial thrombi (André et al., 2002). Monocytic U937 cells are activated by sCD40L-VLA-5 interaction, which results in extracellular-signaling-regulated kinase 1/2 (ERK1/2) pathway activation and gene expression (Léveillé et al., 2007). After CD40L-VLA-5 interaction increased production of pro-inflammatory chemokines and cytokines like IL-1β, IL-6, and MCP-1 were observed (Loubaki et al., 2010; Bian et al., 2018). Furthermore, platelet bound CD40L can induce multimer formation of ultra-large von Willebrand factors (UlvWFs) which are important key players in the initial phase of atherosclerotic plaque genesis. It was shown that CD40L induced UlvWF formation mediates the adhesion of platelets to endothelial cell walls as well as monocyte recruitment (Popa et al., 2018). Taken together, CD40L-integrin signaling as well as CD40L induced UlvWF formation are important mediators of thrombi initialization, thrombi stabilization, inflammation, and macrophage activation.

### 3.2 Classical CD40L-CD40 signaling

CD40L with its classical binding partner CD40 plays a major role in immune responses (Figure 3). CD40 (54 kDa protein) belongs also to the TNF receptor superfamily and is expressed on thrombocytes, immune cells (B cells, dendritic cells, neutrophils, activated T cells, and macrophages) as well as on non-immune cells (endothelial cells, vascular smooth muscle cells, and fibroblasts) (Paulie et al., 1984). Mutations in CD40 or CD40L gene can disrupt its interaction capability and lead to the development of hyper IgM syndrome, which comprises defective class switch recombination, somatic hypermutation, defective T cell priming as well as macrophage and dendritic cell activation which can lead to death by infections due to the inborn immune suppression (La Morena, 2016). In general, CD40 trimerizes and binds to CD40L whereas CD40 itself has no intrinsic capabilities and needs adaptor proteins for signal transduction following its activation (Schönbeck and Libby, 2001). These adaptor proteins are TNF receptor associated factors (TRAFs), which bind to the cytosolic tail of CD40 and activate downstream pathways. CD40 contains two separate binding sites for TRAFs: one distal binding site for TRAF2/3/5 and a proximal one for TRAF6 (Bishop and Hostager, 2001; Bradley and Pober, 2001). A brief summary of the different TRAF molecules (TRAF1-7) and their role in the CD40-CD40L signaling pathway will be given in the following sections.

Heterotrimer formation of TRAF1 (46 kDa), TRAF2 (56 kDa) and CD40 leads to the activation of nuclear transcription factor (NF)-κB and pro-inflammatory p38 mitogen-activated protein kinase (MAPK) pathways (Arron et al., 2002; Wicovsky et al., 2009). TRAF1 protein levels are low in non-activated immune cells, its expression is strongly upregulated in activated lymphocytes and monocytes in an NF-κB dependent manner (Schwenzer et al., 1999; Edilova et al., 2018). It was shown that TRAF1 deficiency led to reduced pulmonary leukocyte recruitment after lipopolysaccharide inhalation presumably by impaired adhesion molecule expression (e.g., ICAM-1 and VCAM-1) as well as impaired chemokine expression (Oyoshi et al., 2007). In high fat treated mice with TRAF1 and low density lipoprotein receptor (TRAF1^−/−^/LDLR^−/−^) deficiency smaller atherosclerotic lesions, which contain less lipids and higher amounts of collagen in comparison to control mice, were observed. In detail, it was shown that the atherosclerotic lesions in TRAF1^−/−^/LDLR^−/−^ mice contain less macrophages and that the adhesion of monocytes is partially inhibited by reduced adhesion molecule expression (e.g., ICAM-1 and VCAM-1) in macrophages and endothelial cells. The differentiation of macrophages was not affected in this mouse model. Furthermore, in patients suffering from acute coronary syndrome a higher TRAF1 blood mRNA expression was observed (Missiou et al., 2010). These findings indicate a pro-inflammatory role of TRAF1 signaling in atherosclerosis.

CD40 forms homodimers with TRAF2 (56 kDa) and lead to an activation of NF-κB, C-Jun N-terminal kinases (JNK) or p38 MAPK signaling (Park et al., 1999; Lalani et al., 2018). TRAF2 deficiency causes chronic inflammation and impaired T cell homeostasis, which is lethal in mice (Lin et al., 2011). It was shown, that TRAF2 protects against autoimmune inflammatory processes and TNF-α induced apoptosis via NF-κB signaling pathway (Yeh et al., 1997). TRAF3 (65 kDa) activates NF-κB signaling after CD40 binding similar to TRAF2 (Devergne et al., 1996; Duckett et al., 1997). TRAF3^−/−^ mice show also a very limited lifespan and a deficiency of TRAF3 protein leads to permanent activation of NF-κB which leads to an multi-organ inflammation as a result of higher cytokine (e.g., IL6 and IL12) levels (Xu et al., 1996; Lalani et al., 2015). Overexpression of TRAF3 prevents CD40-induced endothelial cell activation and inhibits the expression of pro-inflammatory cytokines (Urbich et al., 2001). It seems that TRAF4 does not affect the immune cell development and is not involved in vascular inflammation and/or atherosclerosis (Gissler et al., 2022). TRAF4 is involved in more developmental processes like the formation of the respiratory upper tract (Shiels et al., 2000). TRAF5 (64 kDa) can form homodimers with TRAF3 which force the binding of TRAF5 to CD40. CD40-TRAF5 interaction also activates NF-κB and JNK signaling and as TRAF2 and TRAF3, TRAF5 seems to have also anti-inflammatory properties (Pullen et al., 1998; Leo et al., 1999; Gissler et al., 2021a). For example, TRAF5 shows protective characteristics in a myocardial ischemia/reperfusion injury (Xu et al., 2020). TRAF5 deficiency in an obese mouse model lead to enhanced adipose tissue inflammation compared to control mice which also indicate the anti-inflammatory signaling properties of TRAF5 (Gissler et al., 2021a). In addition, it was shown that TRAF2/3/5 signaling does not affect atherosclerosis or neointima formation (Donners et al., 2008; Lutgens et al., 2010).

In contrast to TRAF2/3/5, TRAF6 (63 kDa) is involved in multiple pro-inflammatory processes (Lutgens et al., 2010). NF-κB signaling is activated through CD40-TRAF6 interaction (Cao et al., 1996). TRAF6 also promotes JNK and p38 MAPK signaling cascades through binding to TRAF2 (Baud et al., 1999). The deficiency of TRAF6 in mice has a serve impact of the immune system: mice lacking TRAF6 show inflammatory cell accumulation in organs, thymic atrophy as well as lack of secondary lymphatic organs (Akiyama et al., 2005; Gissler et al., 2022). The lack of CD40-TRAF6 binding leads to reduced inflammatory cytokine production resulting in the ameliorated progression of atherosclerosis compared to CD40-TRAF2/3/5 deficient mice. Furthermore, it was shown that TRAF6 signaling leads to inflammatory gene expression and polarization of macrophages from regulatory M2 macrophages to the pro-inflammatory M1 phenotype. TRAF6 also promotes monocyte recruitment into atherosclerotic plaques (Lutgens et al., 2010).

**FIGURE 4:**
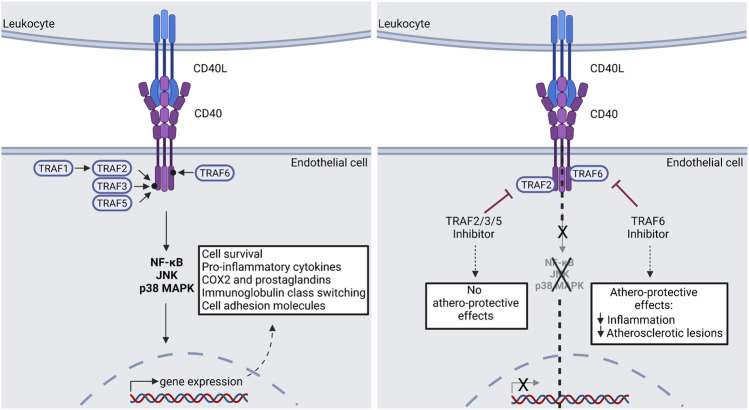
CD40(L) signal transduction *via* TRAF molecules. Down-stream effects of the CD40(L) dyad can be devided into two main groups: The TRAF2/3/5-dependent effects are regarded as athero-protective, while the TRAF6-dependent effects promote atherosclerosis. A potential pharmacological approach is presented in the right panel. Consequently, selective TRAF6-inhibition could be used clinically in order to treat atherosclerosis. TRAF: Tumor Necrosis Factor Associated Factor; JNK: c-Jun N-terminal kinase; p38 MAPK: p38 mitogen-activated protein kinase; NF-κB: Nuclear factor kappa B; COX2: Cyclooxygenase 2. Scheme was summarized from data in (Seijkens et al., 2018). Created with BioRender.com.

As mentioned before, CD40-TRAF signaling can activate separate downstream pathways like NF-κB, C- JNK and/or p38 MAPK (Wajant et al., 2001) (Figure 4). Target gene classes of these pathways are cytokines/chemokines (TNF-α or IL-6), immune receptors (CD40), adhesion molecules (VCAM-1), anti-apoptotic genes (TRAF1/2), enzymes (nNOS, HO-1), stress response genes, growth factors, transcription factors or miRNAs (miR-146) (Schieven, 2005; Waetzig et al., 2005; Mussbacher et al., 2019). In summary CD40L-CD40-TRAF6 signaling seems to be the main driver in macrophage activated pro-inflammatory signaling in atherosclerosis and may therefore promote tissue damage and cell death after MI. In contrast, CD40L-CD40-TRAF2/3/5 signaling seems to have a more regulatory function in the immune system and may contribute to resolution. Regarding TRAF7 (74 kDa) and its role in inflammation or vascular disease nothing has been published so far (Gissler et al., 2022). A more detailed overview concerning the role of all TRAF proteins and their role in vascular inflammation and atherosclerosis has been published (Gissler et al., 2022).

## 4 CD40/CD40L/TRAF signaling cascade—as potential therapeutic target of atherosclerosis

### 4.1 Global inhibition of CD40/CD40L

In general, CD40L deficient mice have a phenotype which is comparable to the human hyper IgM syndrome. While they are exposed to an increased risk of infection, they are viable and fertile (Renshaw et al., 1994). CD40L^−/−^ mice in a hypertension AT-II infusion model demonstrate improved endothelial function, suppression of blood platelet monocyte interaction, improved bleeding time as well as decreased oxidative stress in aorta, heart, and blood (Hausding et al., 2013). Also, in a hyperlipidemia mouse model (16 weeks of a high fat diet) CD40L^−/−^ has a beneficial effect of weight gain and blood cholesterol levels in contrast to control mice. In addition high fat diet associated endothelial dysfunction and vascular oxidative stress, the expression of inflammatory markers and platelet activation is improved in CD40L^−/−^ mice (Steven et al., 2018). In contrast to these data, some studies also address the negative sites of a complete CD40L blocking. It was shown that the lack of soluble CD40L affects the stability of arterial thrombi as frequently ruptured thrombi followed by embolism were observed in CD40L^−/−^ mice in comparison to the control mice. The interaction of CD40L and the transmembrane protein Integrin α_IIb_β_3_ was identified as crucial for thrombi stability (André et al., 2002; Prasad et al., 2003). Monoclonal antibody treatment directed against CD40L in a kidney allograft model in monkeys also resulted in major side effects caused by thromboembolic complications (Kawai et al., 2000). A CD40L antibody (BG9855) was also used in a clinical trial with patients suffering from lupus glomerulonephritis. The trial was terminated after thromboembolic events, which occurred as side effect in study participants (Boumpas et al., 2003). A new CD40L antibody (CDP7657) that lacks a functional Fc region was developed, as the Fc region of the antibody was the supposed mediator of platelet activation, thereby inducing thrombotic events (Shock et al., 2015). A clinical phase 3 trial employing the new antibody under the name Dapirolizumab pegol is currently underway, after safety regarding thrombotic events had been demonstrated earlier (Tocoian et al., 2015; Chamberlain et al., 2017; NCT04294667). Another clinical trial with a humanized monoclonal antibody directed against CD40L (Toralizumab/IDEC-131) is ongoing. The antibody is used in patients suffering from multiple sclerosis and in Phase 1 no thromboembolic events have been reported (Fadul et al., 2021).

CD40 deficient mice are also viable and fertile but show impaired immunoglobulin class switching and germinal center formation (Kawabe et al., 1994). In comparison to CD40L^−/−^ mice, high fat treated CD40^−/−^ mice show no ameliorated tissue inflammation. In contrast, in these animals severe inflammation induced by an increased number of pro-inflammatory CD11^+^F4/80^+^ macrophages within adipose tissue was observed in comparison to control animals. In accordance to these results, in obese CD40^−/−^ mice an aggravated insulin resistance was observed compared to wild-type animals (Chatzigeorgiou et al., 2014). In CD40^−/−^ ApoE^−/−^ mice that lack CD40 as well as the ApoE receptor, thus suffering from a severely impaired capability to handle cholesterol, reduced atherosclerosis progression was observed in comparison to control mice when subjected to a high-fat diet. In detail a reduced plaque size in aortic arches as well as a more fibrotic plaque composition was observed in these mice. In addition, the content of macrophages within the plaques was reduced and the macrophages were polarized to a more anti-inflammatory phenotype in mice lacking CD40 (Lutgens et al., 2010). The main driver of pro-inflammatory CD40 signaling seems to be leukocyte-dependent since impaired macrophage migration and decreased atherosclerosis was observed in leukocyte specific CD40-deficient mice (Lutgens et al., 2010). Several clinical trials in humans regarding CD40 blocking via antibodies are currently ongoing. For example, the monoclonal anti-CD40 antibody ASK1240 is being tested as an immunosuppressant to prevent allograft rejection. Phase 1 was completed and no thromboembolic events or cytokine release syndromes were observed, which allowed further testing in phase 2 trials (Goldwater et al., 2013; Vincenti et al., 2020). The pharmacokinetics and safety of TSK1240 antibody has subsequently been tested in patients suffering of plaque psoriasis (Anil Kumar et al., 2018). Another monoclonal CD40 antibody termed BI 655064 was already tested in phase 1 and 2a as a potential treatment agent for several autoimmune diseases like rheumatoid arthritis, systemic lupus erythematosus or lupus nephritis. So far, the antibody treatment has shown no severe side effects like thromboembolic events (Albach et al., 2018; Visvanathan et al., 2019). More clinical trials are still ongoing regarding the use of monoclonal CD40 antibodies for the treatment of autoimmune diseases like Chron´s disease (Kasran et al., 2005) or systemic lupus erythematosus (Perper et al., 2019).

Taken together it remains unclear whether the pharmacological blockade of CD40(L) as a treatment method for atherosclerosis or other inflammatory diseases will be a viable strategy for the future due to the side effects observed previously, encompassing severe immunosuppression (CD40) or thromboembolisms (CD40L).

### 4.2 Tissue specific inhibition of CD40/CD40L

A systemic blocking of CD40L or CD40 signaling to date does not seem promising as a long-term treatment of atherosclerosis due to the aforementioned unwanted side effects of immunosuppression or thromboembolic events. Therefore, a more targeted therapeutic strategy could lie in the development of cell type specific antibodies or targeted nanopharmaceuticals to avoid negative side effects of a complete or global CD40/CD40L blocking (Duivenvoorden et al., 2019; van de Vyver et al., 2021). Several *in vivo* studies regarding cell type specific deficiency of CD40/CD40L are still ongoing with promising data (Gissler et al., 2021b; Lacy et al., 2021; Bosmans et al., 2022). For example, endothelial-cell specific deletion of CD40 in ApoE^−/−^ mice leads to a more stable plaque phenotype and to reduced leukocyte adhesion compared to control mice when subjected to a high-fat diet. No systemic parameters were affected in these mice and it was also shown that CD40 expression is upregulated in endothelial cells during atherogenesis (Gissler et al., 2021b). Also, T-cell and dendritic cell specific CD40L/CD40 deletion in ApoE^−/−^ mice demonstrate reduced atherosclerotic plaque development as well as reduced plaque inflammation. In these mice a reduced IFN-γ expression as well as impaired T helper 1 polarization was observed, which suggests that CD40L on T cells and dendritic cells is playing an important role in T cell mediated inflammation. It was also shown that in human plasma and atherosclerotic plaques sCD40 as well as sCD40L levels correlate with circulating IFN-γ levels.

These results indicate that in human atherosclerosis, CD40-CD40L signaling is involved in the IFN-γ response which is attributed to T helper 1 cells. As the deletion of platelet-specific CD40L did not affect the size of atherosclerotic plaques or plaque phenotype, the ligand seems to be more involved in athero-thrombosis than atherosclerosis (Lacy et al., 2021). Macrophage specific deletion of CD40 in ApoE^−/−^ mice results in a reduced activated immune profile compared to control mice. In CD40^mac−/−^ mice, reduced atherosclerotic plaque size, reduced necrotic core area, reduced macrophage content and increased collagen production was observed all of which indicate decreased progression of atherosclerosis compared to control mice. This phenotype is probably caused by alternative macrophage activation in atherosclerotic aortas which express a more anti-inflammatory profile than CD40-containing macrophages as well as the prevention of CD40 macrophage polarization towards pro-inflammatory states. Taken together, mice with CD40-deficient macrophages show less inflammation in the atherosclerotic aorta (Bosmans et al., 2022). The cell type specific deletion of CD40 is an emerging field, which could lead to a better understanding of the involvement of CD40-CD40L signaling in inflammation and the progression of atherosclerosis. These data indicate that a cell type specific deletion of CD40(L) could be a promising tool in the future for the treatment of atherosclerosis. Certainly, more research must be done in this regard, but it can be assumed that a partial blocking of CD40 would entail fewer side effects than a global blocking.

### 4.3 CD40-TRAF inhibition

Due to the severe side effects of a complete CD40/CD40L blockade in the first clinical trials further research was done to identify downstream targets for a more selective blockade with reduced side effects. In 2010, it was shown that CD40-TRAF6 signaling but not CD40-TRAF2/3/5 signaling is an important key player in activation of pro-inflammatory immune responses and in the development of atherosclerosis (Lutgens et al., 2010). Therefore, small TRAF stop molecules were created as a new pharmacological drug to target CD40-TRAF6 signaling (Zarzycka et al., 2015). The drug component 6877002 was first tested in an obesity mouse model. The mice were fed with a high fat diet and treated for 6 weeks with the new TRAF6 inhibitor. After treatment, improved insulin sensitivity, reduced CD11b+F480+CD11c+ (M1) macrophages and reduced hepatosteatosis were observed in comparison to the control mice. This indicates that pharmacological TRAF6 inhibition could be a target to prevent obesity related inflammation and metabolic complications (Lutgens et al., 2010; Chatzigeorgiou et al., 2014). Related to this study, pharmacological TRAF6 inhibition was tested in a diabetes mellitus type 2 mouse model for anti-inflammatory properties. The drug treatment lead to reduced body weight gain, enhanced endothelial function, reduced oxidative stress in aorta as well as to reduced inflammatory marker concentration (IL-1β, 3-NT) in blood plasma in comparison to untreated control mice (Steven et al., 2018). Also, this study demonstrates the anti-inflammatory effect of TRAF6 inhibition. An initial study regarding the potential treatment of atherosclerosis with small TRAF6 stops was already performed. There it was demonstrated that TRAF6 stop 6877002 treatment in ApoE^−/−^ mice reduces existing atherosclerosis by decreased macrophage activation and leukocyte recruitment (Seijkens et al., 2018).

In contrast to a general CD40 inhibition, TRAF6 inhibition preserves immunity and does not affect germinal center formation or Ig isotype switching of B cells neither dendritic cell induced co-stimulation of T cells. In addition, selective TRAF6 inhibition prevents pro-inflammatory cytokine secretion of macrophages as well as immune suppressive side effects by preserving CD40-TRAF2/3/5 (anti-inflammatory) signaling and reducing thereby established atherosclerosis. For this reason, TRAF stops represent a promising treatment strategy for atherosclerosis (Seijkens et al., 2018). The TRAF stop treatment was improved by a more targeted macrophage specific TRAF6 inhibition *via* recombinant high density lipoprotein (rHDL) nanotherapy. For this approach TRAF stop 6877002 was incorporated into rHDL nanoparticles. After rHDL-6877002 treatment in ApoE^−/−^ mice as well as non-human primates reduced atherosclerotic plaque size in the aortic roots as well as fewer macrophages within the plaques were observed. The advantage of this targeted nanoparticle treatment in comparison to an untargeted TRAF stop treatment is a reduced dosage application as well as reduced treatment frequency (Lameijer et al., 2018; Seijkens et al., 2018). In general, pharmacological TRAF6 inhibition with component 6877002 was not only analyzed in obesity or atherosclerotic mouse models, but was also found to be a potential therapeutic strategy in other inflammatory or autoimmune diseases like neuroinflammation/multiple sclerosis (Aarts et al., 2017), breast cancer (Bishop et al., 2020), bone cancer (Marino et al., 2022) and in heart failure (Bosch et al., 2019; Marino et al., 2022).

### 4.4 miRNAs related to CD40-TRAF as potential novel therapeutic target of atherosclerosis

**FIGURE 5:**
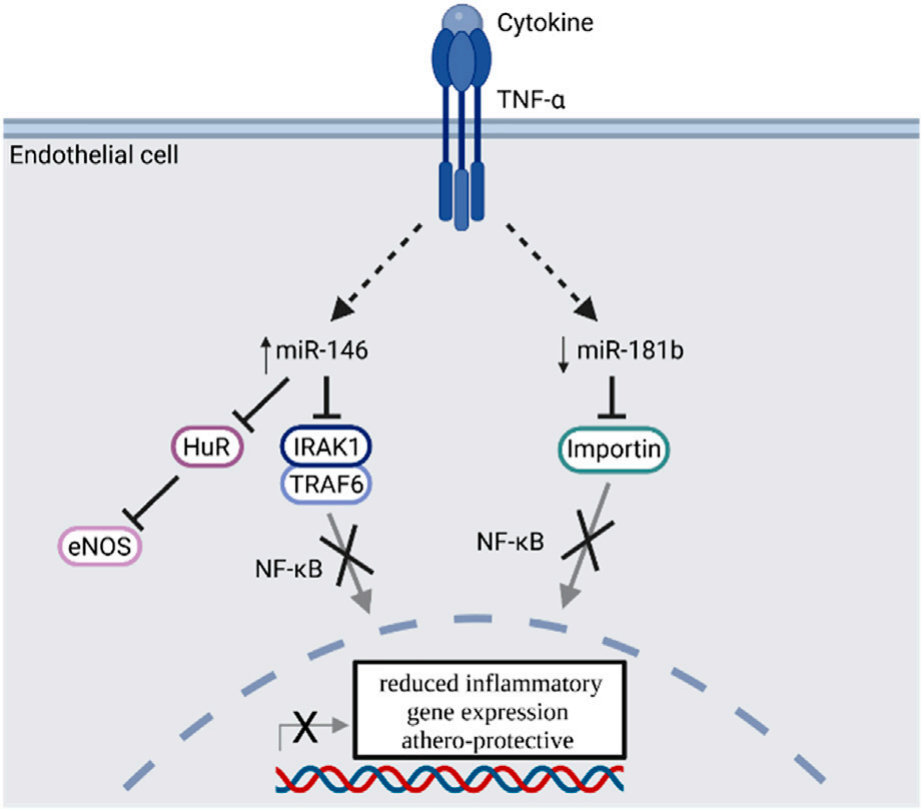
TNF-α signal transduction enhances miR-146 and reduces miR-181b expression, which inhibits NF-κB signaling and is regarded as athero-protective. TNF-α: Tumor necrosis factor alpha; miR: microRNA; HuR: Human antigen R protein; eNOS: endothelial nitric oxide synthase; IRAK1: Interleukin 1 receptor associated kinase 1; TRAF6: TNF receptor associated factor 6; NF-κB: Nuclear factor kappa B. Scheme was summarized from data in (Feinberg and Moore, 2016). Created with BioRender.com.

MiRNA expression is closely linked to many pathological processes like cardiovascular diseases, atherosclerosis and often accompanying disease like obesity or diabetes (Tang et al., 2008; Santovito et al., 2012; Iacomino and Siani, 2017; Wojciechowska et al., 2017). Especially two cytokine responsive miRNAs (miR-146 and miR-181) are described in the literature to be involved in the CD40-TRAF signaling cascade in endothelial cells (Figure 5) (Feinberg and Moore, 2016).

The expression of miR-146a in endothelial cells is induced by TNF-α or IL-1ß and effects the resolution of inflammatory gene expression by regulating NF-κB signaling via targeting TRAF6 and interleukin 1 receptor associated kinase 1 (IRAK1), as well as via the regulation of eNOS expression by targeting HuR in a NF-κB independent manner (Cheng et al., 2013). Overexpression of miR-146a results in reduced inflammatory signaling of endothelial cells. MiR-146 expression is also upregulated in atherosclerotic plaques and is involved in the cholesterol metabolism of macrophages. In oxidized LDL-stimulated macrophages, miR-146 expression leads to a reduced cytokine release and lipid uptake (Yang et al., 2011). Furthermore, miR-146 affects the balance between M1 and M2 macrophages in atherosclerotic plaques (Vergadi et al., 2014). In summary, miR-146 shows anti-inflammatory properties and plays a regulatory role in immune cells like T cells, macrophages and dendritic cells (Feinberg and Moore, 2016). Uniquely in endothelial cells but not leukocytes, miR-181b targets importin-α3 and inhibits NF-κB signaling as well as vascular inflammation (Sun et al., 2014). Furthermore miR-181a is involved in the pathogenesis of atherosclerosis as it targets c-FOS and OPN and reduces the immune-inflammatory response induced by oxidized LDL by decreasing the expression of cell surface proteins like CD40 or CD83 on dendritic cells (Wu et al., 2012). Consequently, pro-inflammatory stimuli lead to a reduced expression of miR-181 *in vitro* and *in vivo* (Sun et al., 2014). In summary miR-146 and miR-181 both regulate parts of the NF-κB signaling and have athero-protective properties. While these properties are elicited by an overexpression of miR-146, atherosclerotic plaques are protected by decreasing miR-181 expression (Figure 5).

## 5 Conclusion

Cardiovascular disease represents a major burden to global health, causing high mortality and reducing quality of life. There are many factors influencing CVD, ranging from rather banal yet impactful factors like lack of sleep and exercise and unhealthy nutrition, to more defined ones encompassing oxidative stress and inflammatory processes. Atherosclerosis and myocardial infarction can be regarded as driven by the latter concepts, evidenced by the degradation of damaged and regeneration of new tissue occurring in a chronologically balanced course during a cardiovascular event via an inflammatory process, termed resolution and remodeling. Crucial within this process are macrophages (M1/M2), which promote both, inflammation as well as resolution, degrading tissue and eliciting regeneration. The CD40(L) dyad is in turn intertwined in the signaling cascades influencing monocytes in their activation and immunological phenotype. A general blockade of CD40(L) signaling has of yet proven impractical regarding the treatment of CVD as the dyad is also involved in thrombus formation and stabilization. In summary, targeting the dyad on specific cells or at specific levels of the involved signaling cascades could be a promising tool in order to regain tissue-homeostasis after MI and subsequent inflammatory processes.
